# Regulated DNA Methylation and the Circadian Clock: Implications in Cancer

**DOI:** 10.3390/biology3030560

**Published:** 2014-09-05

**Authors:** Tammy M. Joska, Riasat Zaman, William J. Belden

**Affiliations:** Animal Sciences, School of Environmental and Biological Sciences, Rutgers, The State University of New Jersey, New Brunswick, NJ 08904, USA; E-Mails: tjoska@gmail.com (T.M.J.); rnz6@scarletmail.Rutgers.edu (R.Z.)

**Keywords:** circadian clock, DNA methylation, facultative heterochromatin, cancer

## Abstract

Since the cloning and discovery of DNA methyltransferases (DNMT), there has been a growing interest in DNA methylation, its role as an epigenetic modification, how it is established and removed, along with the implications in development and disease. In recent years, it has become evident that dynamic DNA methylation accompanies the circadian clock and is found at clock genes in *Neurospora*, mice and cancer cells. The relationship among the circadian clock, cancer and DNA methylation at clock genes suggests a correlative indication that improper DNA methylation may influence clock gene expression, contributing to the etiology of cancer. The molecular mechanism underlying DNA methylation at clock loci is best studied in the filamentous fungi, *Neurospora crassa*, and recent data indicate a mechanism analogous to the RNA-dependent DNA methylation (RdDM) or RNAi-mediated facultative heterochromatin. Although it is still unclear, DNA methylation at clock genes may function as a terminal modification that serves to prevent the regulated removal of histone modifications. In this capacity, aberrant DNA methylation may serve as a readout of misregulated clock genes and not as the causative agent. This review explores the implications of DNA methylation at clock loci and describes what is currently known regarding the molecular mechanism underlying DNA methylation at circadian clock genes.

## 1. DNA Methylation

Methylation on the number 5 carbon of cytosine (5mC) in DNA is found at silenced retrotransposons, imprinted loci and the inactive X chromosome in females [1,2,3,4,5]. DNA methylation is implicated in the etiology of numerous diseases, most notably cancer, and a recent PubMed search revealed over 3000 reviews. Despite this, the role of DNA methylation in cancer remains enigmatic, with hyper-, hypo- or hemi-methylated sequences all potentially contributing to, or being a consequence of, misregulated expression [6,7]. This is due in part to the different regulatory mechanisms by which the methyl group is added to cytosine and further complicated by the discovery that the Tet (ten-eleven translocation) family of proteins can convert 5mC to 5-hydroxymethylcytosine (5hmC) [8]. Maintenance methylation occurs during semiconservative DNA replication that preserves 5mC through mitosis and meiosis, supporting the notion that DNA methylation is the premier epigenetic modification involved in silencing. In contrast, noncoding RNAs (ncRNAs) are involved in *de novo* DNA methylation [9,10], and altered expression of these ncRNAs may impact methylation.

DNA methylation is essential for normal vertebrate development, with genome-wide DNA methylation patterns changing during differentiation [11,12]. Interestingly, although DNA methylation is prevalent in many organisms, it is extremely rare or absent in some metazoans, like *Drosophila* and *C. elegans*, indicating that transcription factors and underlying histone modification(s) are sufficient for the developmental program [13,14]. DNA methylation only appears to be essential for viability in higher vertebrates, providing the counter argument that the complexity of the organism’s genome and the need for carefully controlled and timed differentiation dictates the need for DNA methylation-mediated regulatory function. This ongoing debate only serves to pique curiosity and provide continued interest in a modification that now appears to be more dynamic than originally thought. Thus, DNA methylation, once considered a static permanent modification, is dynamic and may have context-dependent roles.

## 2. Genome-Wide Analyses

Genome-wide analysis of DNA methylation has revealed a landscape far more involuted than once imagined. In mammals, 5mC was originally thought to occur predominantly at CpG dinucleotides, and studies in embryonic stems (ES) cells using reduced representation bisulfite sequencing (RRBS) revealed a bimodal distribution that correlates with the underlying histone modifications [12]. Later genome-wide studies in ES cells at base-pair (bp) resolution demonstrated that approximately one-quarter of all 5mC occurs in a non-CpG context, but is lost upon differentiation [15]. However, the mammalian brain appears to be the notable exception, where non-CpG methylation persists [16]. How this impacts the association of MeCP2 or other methyl DNA binding proteins with chromatin needs additional work. These, as well as other genome-wide studies indicate wide-spread distribution of 5mC and demonstrate the dynamic nature of DNA methylation. The metamorphic nature of DNA methylation is not restricted to development, and transient, dynamic 5mC has been observed at circadian clock genes in *Neurospora* and mammals [17,18]. DNA methylation is also detected at clock genes in *Arabidopsis*, but its role in the clock has not been extensively examined [19]. In *Drosophila*, DNA methylation is extremely rare, and there are no reports at clock genes [13]. Therefore, this review is mostly limited to the *Neurospora* and mammalian circadian systems.

## 3. The Circadian Clock

**Figure 1:**
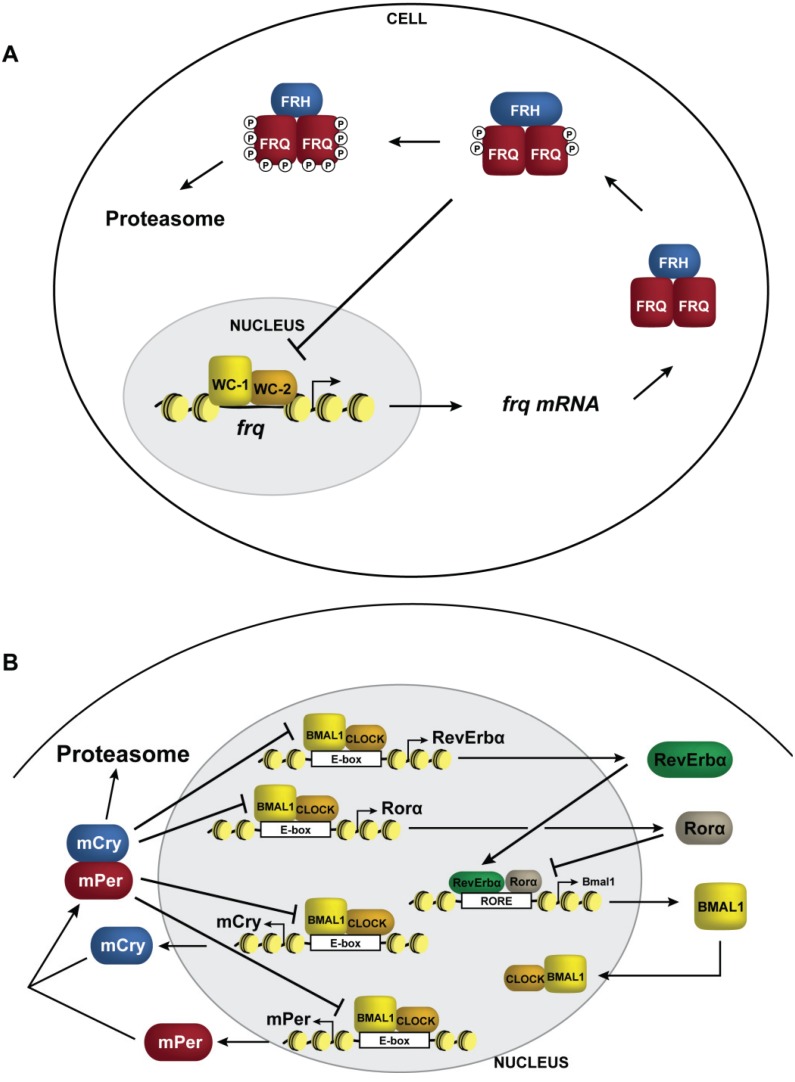
Transcriptional negative feedback in the clock. Schematic representation of circadian negative feedback in (**A**) *Neurospora* and (**B**) mammals. The negative elements are shown in red (FRQ and mPer) and blue (FRH and mCry), and the positive elements are shown in yellow (WC-1 and BMAL1) and orange (WC-2 and CLOCK). For simplicity, only a single *Per* gene and single *Cry* gene were included. Rorα and Rev-Erbα, which form a second interconnect loop in mammals regulating rhythms in *Bmal1* expression, are shown in beige and green, respectively.

The circadian clock is an entrainable, free-running, temperature-compensated, anticipatory system that allows organisms to prepare for daily changes in the environment that arise from the Earth’s rotation [20,21,22,23,24,25]. In this capacity, it controls, among others, daily metabolism and subsequent activity rhythms in mammals and insects, asexual spore development in *Neurospora* and leaf movement in *Arabidopsis* [26,27,28,29,30,31]. The clock rhythm in gene expression persists in part due to time-of-day chromatin-associated events that facilitate the core transcriptional negative feedback loop. The physiological output of the circadian rhythm results from oscillations in genome-wide expression of *clock controlled genes* (*ccg*) that range from greater than 10% of the genome in mammals to as much as a third of the genome in *Arabidopsis* [32,33,34,35]. Moreover, oscillating transcripts differ based on cell/organ type with a subset exhibiting one-quarter (6 h) and one-half (12 h) harmonics [34,36]. There is an extensive number of molecular events that control global changes in *ccg* expression, and a large focus in the molecular biology era has centered on understanding the interconnected transcription/translational feedback loops (TTFL) [37,38]. As our knowledge continues to grow, it is clear that the circadian clocks from fungi to mammals consist of a transcriptional negative feedback loop, where heterodimeric, PAS domain-containing transcription factors drive the expression of negative elements that feedback, with delays, to inhibit their own expression (Figure 1). In two widely-studied clock systems, the activators consist of WHITE COLLAR (WC)-1:WC-2 in *Neurospora* and CLOCK:BMAL1 in mammals [39,40,41,42]. The negative elements are FREQUENCY (FRQ) and FRQ-interacting RNA helicase (FRH) in *Neurospora* and PERIOD (Per) 1, 2 and 3 and cryptochrome (Cry) 1 and 2 in mammals [43,44,45,46,47,48,49]. In addition, mammals have a secondary loop where Rev-Erbα and Rorα coordinate to control rhythms in BMAL1 expression [50,51]. Once the negative elements are translated, they receive extensive post-translational modifications that serve to regulate nucleo-cytoplasmic shuttling, which allows them to block their own expression [52,53,54,55,56,57]. Eventually, they become terminally modified with extensive phosphorylation, causing them to be destabilized, ubiquitinated and degraded by the proteasome. Many of the numerous reviews referenced above describe the molecular mechanism of the circadian clock, the corresponding covalent modifications and how they impact the central oscillator and output. The focus of this review is on DNA methylation and chromatin in the circadian clock.

## 4. The Clock and Chromatin

The combined nature of the transcriptional negative feedback loop and genome-wide oscillations in *ccg* expression requires coordinated control of chromatin and the genome structure [58,59,60]. In this capacity, it should not be surprising that the circadian field has seen a boom in discoveries involving chromatin-associated enzymes involved in clock function. Moreover, Per complexes contain a variety of interacting partners with known catalytic activity toward chromatin [61,62,63]. The chromatin enzymes identified thus far can be grouped into four classes; activating, repressive, amplitude-modulating or phase-modulating, based on their occurrence and the resulting phenotype. Moreover, the enzymes only tell a partial tale, because circadian-regulated metabolism provides substrates in the form of S-adenosyl methionine (SAM/AdoMet), nicotinamide adenine dinucleotide (NAD^+^) and acetyl co-enzyme A (Acetyl-CoA) utilized by methyltransferases, histone deacetylases and acetyltransferases, respectively, providing an additional interconnected feedback network that helps controls the amplitude, phasing and underlying rhythm [64,65,66,67].

There are a significant number of coincident histone modifications that change over the circadian cycle, and in some cases, the corresponding histone-modifying enzymes have been identified. Histone H3 serine 10 (H3S10) phosphorylation was the first chromatin mark implicated in circadian-regulated gene expression, and it increases in the mouse SCN neurons when exposed to light at night [68]. Later studies documented rhythms in acetylation of histone H3 in *mPer1*, *mPer2* and *Cry1* promoters, with the peaks occurring during the transcriptionally active phase [69,70]. Rhythms in acetylated histones presumably occur as a direct result of the transcriptional activator, CLOCK. Affinity purification of CLOCK indicates that it associates with the ubiquitous KAT3B, p300 [69] and possesses its own catalytic acetyltransferase activity [71]. In mouse embryonic fibroblasts (MEFs), CLOCK has also been shown to interact with the KMT2, MLL1 (mixed-lineage leukemia) and MLL1 and catalyzes the methylation of H3K4 [72]. Collectively, these data indicate that components of the biological clock can direct chromatin modifications to help maintain rhythms. The observations of oscillations in histone acetylation naturally lead to the identification of a growing list of HDACs that include HDAC1 and HDAC2 (part of the Sin3B complex) [73] and the NAD-dependent HDAC, SIRT1 [74,75]. In addition, polypyrimidine tract-bind protein-associated factor (PSF) is in a complex with PER and recruits HDACs associated with Sin3A [62].

Methylation and demethylation of histones also occurs coincident with circadian-regulated gene expression. However, some of the details surrounding many of the enzymes are still underdeveloped. It has been demonstrated that during the repressive phase, there is di- and tri-methylation of H3K27 at *mPer1* and *mPer2*, which is dependent on the Polycomb group protein, EZH2 (KMT6) [76]. In studies performed on mouse livers, H3K9Ac is rhythmic in the *D-element binding protein* (*Dbp*) gene (a high-amplitude *ccg*), and studies in MEFs demonstrate a rhythm in H3K4me3 that is catalyzed by MLL1 (mixed-lineage leukemia) [72,77]. However, ChIP-seq data examining genome-wide oscillations in H3K4me3 indicate that it is prevalent during the repressive phase of the circadian cycle, and this is consistent with studies in *Neurospora*, indicating that the KMT2, SET1, is needed for efficient negative feedback inhibition [78]. Interestingly, JARID1a, a known H3K4 lysine demethylases (KMD), does not appear to have any effect on H3K4me3, but instead inhibits HDAC1 recruitment [79]. However, overexpression of JARID1b and JARID1c can potentially reverse H3K4 methylation, but this has not been examined in great detail [79].

In addition to methylation of lysine 4, there exists rhythmic facultative heterochromatin consisting of H3K9me2 and HP1 binding at *Dbp*, *Per1* and *Per2* during the repressive phase, and presumably, this occurs through the association of Suv39h and HP1γ with PER2 [63,77]. Lysine-specific demethylase (LSD1), which can remove methyl groups from H3K4 and/or H3K9, associates with CLOCK and BMAL1 and could potentially serve to reverse H3K9 methylation. However, the mutant *Lsd1* used in this study did not affect H3K9me3 levels [80]. In addition, both the mammalian and *Arabidopsis* circadian clocks utilize the histone demethylase JMJD5 to demethylate H3K36 [81]. Overall, there appears to be ordered recruitment of activating and repressive modifications that ultimately generate facultative heterochromatin on the circadian time-scale.

In *Neurospora*, much of the research has been focused on ATP-dependent chromatin-remodeling enzymes that coordinately control the chromatin architecture at *frq*. CLOCKSWITCH (CSW-1) and CATP (CLOCK ATPase) remodel chromatin at the C-box element, functioning during the repressive and activation phases, respectively [82,83]. A chromodomain helicase DNA binding family member, CHD1, appears to be required for activation and has the interesting phenotype of causing an increase in heterochromatic spreading at *frq* [17]*.* The cause and implications of this DNA hypermethylation phenotype are still unknown, but possible reasons are discussed below.

**Figure 2:**
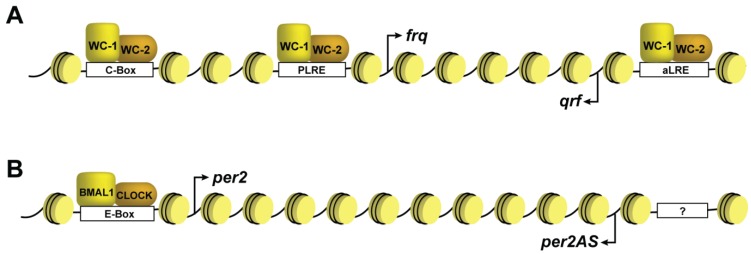
Schematic of the clock genes. Illustration of (**A**) the *frq* gene in *Neurospora* and (**B**) the *Per2* gene in mammals, shown as the typical “beads on string” with nucleosomes shaded yellow. The figure illustrates the sense and antisense transcripts that originate from both loci. The cis-acting sequences are shown as boxes on the DNA and labeled accordingly. The “?” within the *Per2AS* promoter indicates that the transcription factors responsible for expression are still unknown. Note that these representations are not drawn to scale.

A paradoxical observation in circadian research is the finding of antisense transcripts that originate from the *frq* gene in *Neurospora* and all three *Period* genes in mammals [58,59,84] (Figure 2). Moreover, overlapping sense/antisense transcripts have been observed in *Arabidopsis* with a specific pair displaying antagonistic circadian expression patterns [85]. In general, these circadian natural antisense transcripts (NATs) are expressed antiphasic to the endogenous protein coding clock genes and in the case with the *frq* antisense *qrf*, expression is driven by the same transcriptional activators [84,86]. This incongruous expression confounds simple linear models of negative feedback and necessitates a molecular switch directing transcript synthesis. In doing so, there must also be amplitude modulation, since the NATs are lower abundance relative to their sense counterparts. Thus far, our findings and those of others indicate that the antisense transcript is involved in DNA methylation and rhythmic facultative heterochromatin formation at *frq*; both of which are needed for proper phasing [17,87].

## 5. DNA Methylation in *Neurospora*

**Figure 3:**
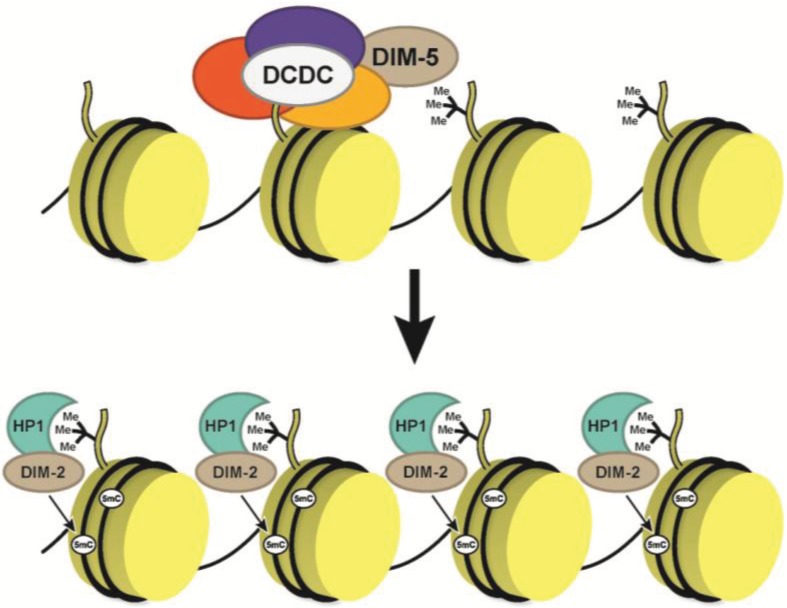
DNA methylation in *Neurospora*. The current model for DNA methylation in *Neurospora* suggests a multistep process that starts with DIM-5 recruitment and H3K9 methylation mediated by the DCDC complex. Once the H3K9me3 is established, HP1 in association with DIM-2 and binds to H3K9me3 via its chromodomain, and then, DIM-2 catalyzes 5mC.

Before exploring RNA-induced DNA methylation at clock genes that is mediated in part by the convergent transcripts, it is useful to review general DNA methylation in *Neurospora* (Figure 3)*.* *Neurospora* has little, if any, gene redundancy, forcing many of the methylation enzymes to function both at repetitive regions and at promoters. Methylation in *Neurospora* occurs largely at relics of repeated-induced point mutations (RIP’ed regions), whose origins were likely transposable elements [88]. DNA methylation requires the DNMT, DIM-2 (defective in methylation), heterochromatin protein 1 (HP1) and a multisubunit complex, DCDC (DIM-5,/-7/-9, CUL4/DDB1 complex) [89,90,91,92,93]. A core subunit of DCDC is the Histone H3 lysine 9 (H3K9) methyltransferase DIM-5 (KMT1) that is needed for mono- (H3K9me1), di- (H3K9me2) and trimethylation (H3K9me3) [94,95]. Current models suggest that DCDC is recruited to RIP’ed regions by DIM-7, although how this recruitment occurs is still unknown. Then, DIM-5, in association with CUL4/DDB1/DIM-9, catalyzes H3K9me3 [91]. DIM-2 is associated with HP1 and HP1 binds to H3K9me3 via its chromodomain, facilitating the subsequent DNA methylation [96]. Interestingly, DIM-2 has little to no effect on growth and only minor phenotypes, compared to DIM-5 or the *hpo* mutant (gene encoding HP1), suggesting that DNA methylation is likely ancillary relative to the H3K9me3 and HP1 binding.

## 6. Dynamic DNA Methylation at Circadian Clock Genes

It is now unequivocal that circadian disruption results in substantial health consequences, and numerous studies indicate shift workers suffer a higher incidence of cancer [97,98,99]. Because anomalous DNA methylation correlates with cancer, it was natural to explore DNA methylation at clock genes. Some early studies found changes in promoter 5mC at *Per1*, *Per2* and *Per3* in breast cancerous tissues relative to surrounding non-cancerous tissue [100]. Since then, aberrant DNA methylation patterns have been found in every core clock gene in a variety of malignancies, and in most instances, the methylation status correlates with expression [101,102,103,104,105]. Methylation defects are also found at *Clock* (hypomethylated) and *Cry*2 (hypermethylated), as well as pathways involved in DNA recombination and repair in shift-workers [106]. Whether or not these changes in methylation are a cause or an effect is still unknown. However, data from *Neurospora* suggest that DNA methylation differences are an effect of clock misregulation, because cells that contain a dysfunctional clock have aberrant 5mC in *frq*.

The molecular mechanism underlying DNA methylation at clock genes in mammals and why DNA methylation at these loci is altered in cancer cells is largely unknown. In contrast, the molecular mechanism of clock gene DNA methylation is better understood in the *Neurospora* circadian system, in part due to *Neurospora*’s unique characteristics. Unlike mammals, where all the DNMT are essential for viability, loss of *dim-2* in *Neurospora* has no discernable effect on cell growth and only minor effects on the clock [17]. Part of this may reside in the simple genomic makeup of *Neurospora*, because it only has a small subset of genes that contain DNA methylation and substantially less repetitive DNA where 5mC is normally prevalent [87,88].

The discovery of DNA methylation at the central clock gene *frq* arose serendipitously while characterizing the ATP-dependent chromatin-remodeling enzyme, CHD1 [17]. Common assays used to examine chromatin remodeling are restriction site accessibility experiments or MNaseI assays, both of which are reliant on restriction endonuclease cleavage followed by southern blot analysis. In MNaseI experiments designed to unravel the remodeling activity of CHD1, methyl-sensitive restriction enzymes had a stark reduction in activity. Further analysis revealed that the strains lacking *chd1* had a hyper DNA methylation defect that caused spreading of the methylated regions beyond what was normally observed in an isogenic wild-type strain. Characterization of the DNA methylation indicated that it requires a functional clock (including both FRQ and FRH), the NAT *qrf* and WCC-mediated gene expression [17]. This supports the claim that changes to DNA methylation at clock genes often observed in cancer cells may arise because the underling clock is no longer functioning properly.

**Figure 4:**
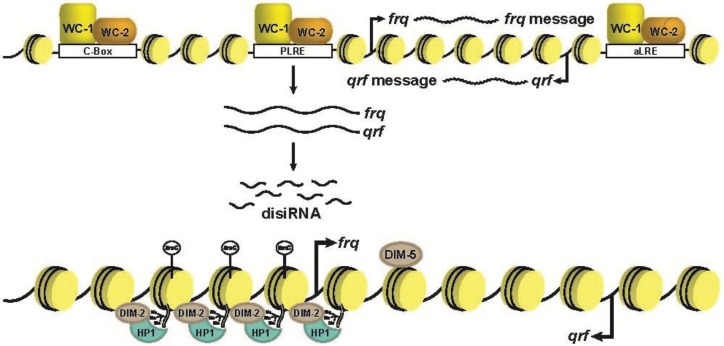
DNA methylation at the *frq*. The molecular mechanism of *de novo* DNA methylation in *Neurospora* at *frq*. The sense antisense pair gives rise to disiRNA that promote H3K9me3. Once the H3K9me3 is established, there is subsequent binding of HP1 and DNA methylation mediated by DIM-2.

The requirement of *qrf* for normal DNA methylation at *frq* is a strong indication of a mechanism analogous to RdDM/RNAi-mediated heterochromatin formation. This notion was further supported by the findings that convergent transcription of *frq*/*qrf* produces dicer-independent siRNA (disiRNA), and DNA methylation is present at all of the approximate 50 disiRNA loci [87,107]. Work recently submitted by our laboratory indicates that *qrf* expression supports *frq* expression by creating a more permissible chromatin state. However, once the level of *qrf* reaches a threshold, it stimulates DIM-5-mediated facultative heterochromatin formation, which includes H3K9me3 and HP1 binding, and this is needed for the terminal DNA methylation generating a repressive environment (Figure 4) [108,109]. Of special note, alluding to the possible function of DNA methylation, is the observation that H3K9me3 levels at *frq* are not preserved in the DNMT mutant *dim-2* [87]*.* This suggests that DNA methylation may serve as a terminal modification that inhibits H3K9-specific demethylation. In other words, in the absence of the terminal DNA methylation modification, the underlying histone modifications are either actively removed or not maintained as effectively. Moreover, this finding is not unique to *Neurospora* and has been observed in *Arabidopsis*, leading us to speculate that in some instances, DNA methylation may serve as a terminal modification to prevent the removal of the underlying H3K9me3 [110,111,112]. This hypothesis can be easily examined in *Neurospora* (or *Arabidopsis*) through close examination of a double mutant containing the both H3K9 *KDM* and DNMT.

## 7. DNA Methylation, the Circadian Clock and Cancer

The discovery that the clock can impart control on some DNA methylation piques curiosity underlying the connections between the clock, DNA methylation and cancer. There are many *ccg* in key regulatory processes connected with cancer progression, including DNA repair, cell cycle, apoptosis and the developmental program [34]. In fact, many tumor suppressors and oncogenes are under circadian control, and *Per* genes function as tumor suppressors [113]. Once such *ccg* is *cMyc*, which is misregulated in the absence of *Per*2, and mice lacking *Per*2 expression are cancer prone [113]. Reciprocally, major tumor suppressors, like p53, are under circadian control, and in the case with p53, it directly regulates *Per2* expression [114]. Paradoxically, overall clock gene expression is typically impaired in cancers, creating a scientific “catch-22” where cause-and-effect is blurred, but cellular homeostatic interconnected feedback among the clock genes (including antisense transcripts), key regulators (tumor suppressor and oncogenes), neuroendocrine hormones and epigenetics reign supreme. Support for this notion comes from the anti-proliferative effect of both melatonin and β-endorphin on breast cancer [115,116].

Ultimately, defects in the clock likely contribute to cancer due to misregulation of a variety of critical cell-physiological processes, and oncogenic mutations affect cross-talk with clock expression. This notion likely underlies at least some of the DNA methylation alterations observed in cancer and is supported by a comparison of two independent reports. First, circadian transcriptional profiling revealed oscillations in lincRNA and NATs that coincided with rhythmic histone modifications, but DNA methylation remained relatively stable [59]. Second, and in contrast, altered light reprogramming to 22-h day caused global changes to DNA methylation and increased expression of *Dnmt3l* [18]. These reports, combined with studies in *Neurospora*, collectively indicate that DNA methylation is dynamic, but relatively stable, unless the clock is perturbed or broken*.* Considering *Period* NATs, other rhythmic NATs and lincRNA that comprise the >95,000 lncRNA transcripts in humans [117], all of which have the potential to alter chromatin and DNA methylation throughout the genome, then it is not surprising that misregulation of clock genes found in cancers may cause changes in DNA methylation. Ergo, the ncRNA transcriptome that has a prominent role in the RdDM is most certainly altered when the circadian rhythm is disrupted, and this is potentially responsible for the changes in DNA methylation.

## 8. Misregulated Genes and DNA Methylation: The Chicken or the Egg?

The notion that small ncRNAs, such as piRNA, or in the case with *Neurospora*, disiRNA, are involved in *de novo* DNA methylation raises a circular question that may be at the heart of our understanding of DNA methylation. If the genetic units that give rise to the small noncoding are somehow misregulated, then this most certainly will cause aberrant DNA methylation. Consequently, DNA methylation may be a terminal readout of misregulated gene expression, and this may account for the changes in 5mC with cell passage [12]. Concrete support for this is limited, but data showing DNA methylation at *frq* is affected in every clock mutant tested, and that H3K9me3 is not maintained at *frq* in the absence of the DNMT, DIM-2, suggests that DNA methylation is needed, at least in this instance, to prevent reversion to a more transcriptionally permissive state. This question is inherently difficult in other systems, due to either the lack of DNA methylation, as is the case with *Drosophila* or *C. elegans*, or the viability issues that occur in mammals. However, non-circadian day lengths do change DNA methylation states in mice [18]. Contrarily, if DNA methylation is a pinnacle modification in gene regulation, either hypo- or hyper-methylation would, by default, cause changes in gene expression. In addition, because DNMT is essential for viability in vertebrates, there are likely enormous pleiotropic effects that arise in silencing experiments, further complicating an already perplexing biological phenomenon. When considering both arguments, one must also consider the notion of context-dependent events, especially in the circadian system, where gene expression may persist, even in methylated promoters [17,59].

Thus, results indicating defects in DNA methylation at clock genes in cancer cells may be more indicative of defective clock gene expression, because any time you perturb the clock, the methylation status will naturally be different than in normal cells. Thus, DNA methylation may not be the causative agent of defective regulation, but instead a readout.
